# Thrombin-Induced COX-2 Expression and PGE_2_ Synthesis in Human Tracheal Smooth Muscle Cells: Role of PKCδ/Pyk2-Dependent AP-1 Pathway Modulation

**DOI:** 10.3390/ijms242015130

**Published:** 2023-10-13

**Authors:** Chien-Chung Yang, I-Ta Lee, Yan-Jyun Lin, Wen-Bin Wu, Li-Der Hsiao, Chuen-Mao Yang

**Affiliations:** 1 Department of Traditional Chinese Medicine, Chang Gung Memorial Hospital at Taoyuan, Taoyuan 333008, Taiwan; r55161@cgmh.org.tw; 2 School of Traditional Chinese Medicine, College of Medicine, Chang Gung University, Taoyuan 333323, Taiwan; 3 School of Dentistry, College of Oral Medicine, Taipei Medical University, Taipei 110301, Taiwan; itlee0128@tmu.edu.tw; 4 Ph.D. Program for Biotech Pharmaceutical Industry, China Medical University, Taichung 406040, Taiwan; u108306002@cmu.edu.tw; 5 School of Medicine, Fu Jen Catholic University, New Taipei City 242062, Taiwan; wenbin@mail.fju.edu.tw; 6 Graduate Institute of Biomedical and Pharmaceutical Science, Fu Jen Catholic University, New Taipei City 242062, Taiwan; lidesiao@livemail.tw

**Keywords:** cyclooxygenase, prostaglandin E_2_, thrombin, resveratrol, human tracheal smooth muscle cell, airway inflammation

## Abstract

In this study, we confirmed that thrombin significantly increases the production of COX-2 and PGE_2_ in human tracheal smooth muscle cells (HTSMCs), leading to inflammation in the airways and lungs. These molecules are well-known contributors to various inflammatory diseases. Here, we investigated in detail the involved signaling pathways using specific inhibitors and small interfering RNAs (siRNAs). Our results demonstrated that inhibitors targeting proteins such as protein kinase C (PKC)δ, proline-rich tyrosine kinase 2 (Pyk2), c-Src, epidermal growth factor receptor (EGFR), phosphatidylinositol 3-kinase (PI3K), or activator protein-1 (AP-1) effectively reduced thrombin-induced COX-2 and PGE_2_ production. Additionally, transfection with siRNAs against PKCδ, Pyk2, c-Src, EGFR, protein kinase B (Akt), or c-Jun mitigated these responses. Furthermore, our observations revealed that thrombin stimulated the phosphorylation of key components of the signaling cascade, including PKCδ, Pyk2, c-Src, EGFR, Akt, and c-Jun. Thrombin activated COX-2 promoter activity through AP-1 activation, a process that was disrupted by a point-mutated AP-1 site within the COX-2 promoter. Finally, resveratrol (one of the most researched natural polyphenols) was found to effectively inhibit thrombin-induced COX-2 expression and PGE_2_ release in HTSMCs through blocking the activation of Pyk2, c-Src, EGFR, Akt, and c-Jun. In summary, our findings demonstrate that thrombin-induced COX-2 and PGE_2_ generation involves a PKCδ/Pyk2/c-Src/EGFR/PI3K/Akt-dependent AP-1 activation pathway. This study also suggests the potential use of resveratrol as an intervention for managing airway inflammation.

## 1. Introduction

The contraction of tracheal smooth muscle in response to various proinflammatory mediators, neurotransmitters, and external agents plays a crucial role in regulating respiratory ventilation. This process is essential for maintaining homeostasis and contributes significantly to the pathogenesis of lung-related conditions [1]. In pulmonary inflammatory diseases like asthma and acute lung injury, the arachidonic acid (AA) pathway assumes a pathological role through generating various metabolites, including prostanoids, leukotrienes, dihydroxyeicosatrienoic acids, and eicosatetraenoic acids [2,3]. Additionally, the cyclooxygenase (COX)-2/prostaglandin E_2_ (PGE_2_) signaling pathway may contribute to the adaptation of airways to exercise under normal physiological conditions [4]. Prostaglandins (PGs), bioactive mediators generated from AA through the enzymatic activity of COX, include constitutive COX-1 and inducible COX-2 [5]. The increased synthesis of PGE_2_ depends on the up-regulation of COX-2 expression in human tracheal smooth muscle cells (HTSMCs) [5]. PGE_2_ can have diverse effects on inflammatory airway diseases. For instance, it is implicated in lung inflammation, and its levels correlate with the severity of airflow obstruction in patients with chronic obstructive pulmonary disease (COPD) [6]. PGE_2_ helps maintain the tracheal muscle tone through activating EP1 and EP2 receptors [7]. Additionally, it exhibits anti-inflammatory properties through the EP4 receptor [8]. Moreover, in COPD fibroblasts, the COX-2/PGE_2_ signaling pathway regulates inflammation and senescence [9]. These findings highlight the significance of the COX-2/PGE_2_ signaling pathway in tracheal smooth muscle cells and its potentially significant role in lung disorders through mediating airway inflammation.

Thrombin is widely recognized as a crucial protease in the coagulation cascade and homeostasis. Additionally, thrombin exerts pleiotropic effects, including inflammation, cell proliferation, and the regulation of vascular tone [10]. Previous literature indicates elevated thrombin production in patients with COPD and asthma compared to healthy individuals [11,12]. Thrombin’s involvement has also been documented in lung cancer and lung fibrosis [11,12,13,14]. One potential contributing factor to thrombin-induced lung diseases might be the up-regulation of COX-2 expression and the synthesis of PGE_2_ [15,16,17]. In our previous studies, we confirmed that thrombin induces COX-2 expression through distinct signaling pathways in HTSMCs [18], primary human cardiomyocytes [19], and human lung fibroblasts [20]. However, a comprehensive understanding of the mechanisms governing thrombin-regulated COX-2 expression in HTSMCs is still lacking.

Protein kinase C (PKC)δ, the first novel PKC isoform discovered through the screening of mammalian cDNA libraries, shares structural similarities with classical/conventional PKC isoforms. It exhibits ubiquitous expression across various cells and tissues. A prior study highlighted that PKCδ, when activated through different pathways, plays pivotal roles in fundamental cellular functions like growth, differentiation, apoptosis, and migration [21]. Focal adhesion tyrosine kinases, FAK, and proline-rich tyrosine kinase 2 (Pyk2) are strategically positioned as vital mediators in signaling pathways governing cell migration, proliferation, and survival [22,23]. Thrombin has been identified as a mediator triggering the activation of PKCδ and Pyk2 in different cell types [23,24]. Wrenn demonstrated that exposure of rat pancreatic acinar cells to phorbol myristate acetate (PMA), a phorbol ester activating PKC, led to increased phosphorylation of PKCδ and Pyk2 [25]. Additionally, c-Src, a common element participating in the cross-talk between cytoplasmic protein tyrosine kinases and receptors, has been implicated in COX-2 expression in A549 cells [26]. Previous studies have indicated that c-Src regulates thrombin-triggered transactivation of platelet-derived growth factor receptor (PDGFR) and epidermal growth factor receptor (EGFR), further intensifying inflammatory responses [23,27]. Recent research has suggested the crucial role of numerous components in the phosphatidylinositol 3-kinase (PI3K)/protein kinase B (Akt) pathway in the expression and activation of inflammatory mediators, inflammatory cell recruitment, immune cell function, airway remodeling, and corticosteroid insensitivity in chronic inflammatory respiratory diseases [2]. Our prior study demonstrated that thrombin up-regulates COX-2 expression and PGE_2_ release through PI3K/Akt activation in human neonatal ventricular cardiomyocytes [28]. The COX-2 promoter contains multiple binding sequences for various transcription factors, including activator protein-1 (AP-1) [29]. AP-1 is a diverse group of dimeric transcription factors comprising Jun, Fos, and Jun dimerization partners (JDPs) and ATF subunits. Among the AP-1 subunits, c-Jun stands out as the most significant transcriptional activator during inflammation [2]. AP-1 activation is mediated through various signaling pathways, such as PKCδ [30], Pyk2 [23], c-Src [23], or PI3K/Akt [23]. Despite studies demonstrating that COX-2 induction is regulated by diverse signaling components and transcription factors, it remains unclear whether these pathways are involved in COX-2 expression and PGE_2_ release in HTSMCs challenged with thrombin. Therefore, our objective is to unveil the molecular mechanisms governing the thrombin-regulated COX-2/PGE_2_ signaling pathway in HTSMCs.

Resveratrol, a natural phytoalexin, possesses diverse biological functions, including anti-oxidative and anti-inflammatory properties [31]. Emerging evidence suggests its potential benefits in various human diseases, particularly cardiovascular and respiratory disorders [32,33]. Recent reports have indicated that resveratrol might be a promising candidate for managing inflammatory conditions through modulating the expression of proinflammatory mediators through signaling components in different cell types [34,35,36,37,38]. However, it remains unclear whether resveratrol can influence thrombin-induced inflammatory responses. To address these questions, experiments were conducted to examine the impact of thrombin on COX-2 expression and PGE_2_ release as well as the anti-inflammatory effects of resveratrol in HTSMCs. The findings reveal that increased COX-2 expression and PGE_2_ release in thrombin-challenged HTSMCs are mediated through a PKCδ/Pyk2/c-Src/EGFR/PI3K/Akt-dependent AP-1 activation pathway. Furthermore, resveratrol attenuates the activation of Pyk2, c-Src, EGFR, Akt, and c-Jun, thereby suppressing COX-2 expression and PGE_2_ release induced by thrombin in HTSMCs. Understanding the mechanisms driving COX-2 induction by thrombin in tracheal smooth muscle could open avenues for novel therapeutic interventions in the management of respiratory diseases.

## 2. Results

### 2.1. Thrombin Induces COX-2 Expression and PGE_2_ Release via PKCδ

**Figure 1:**
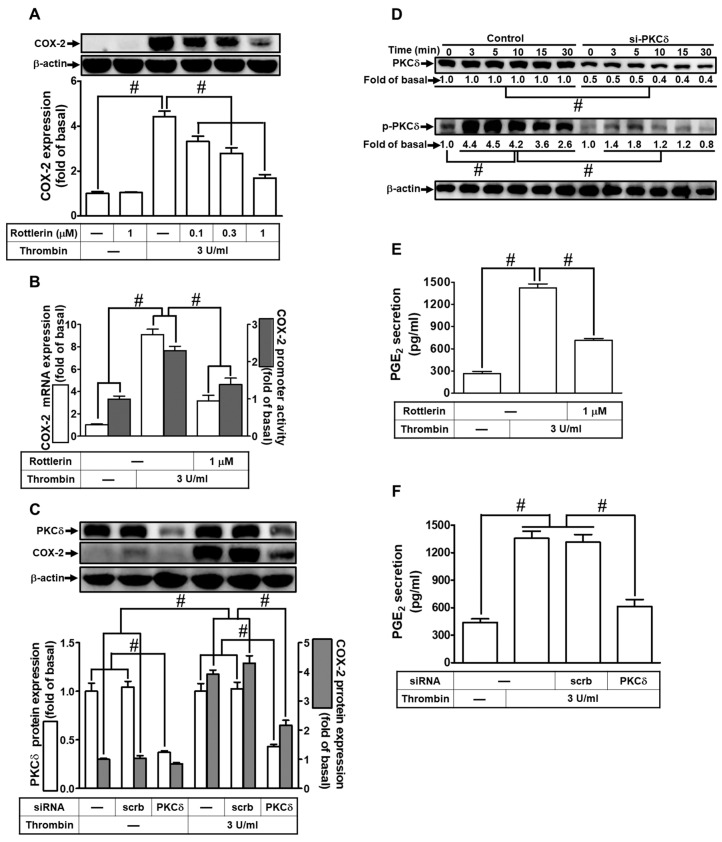
Thrombin induces COX-2 expression via PKCδ. (**A**) Cells were pretreated with Rottlerin (0.1, 0.3, and 1 µM) for 1 h and then incubated with 3 U/mL thrombin for 6 h. COX-2 and β-actin protein levels were determined via Western blot. (**B**) Cells were pretreated with Rottlerin (1 µM) for 1 h and then incubated with 3 U/mL thrombin for 4 h. COX-2 mRNA levels were determined via real-time PCR (open bars), and COX-2 promoter activity was assessed via promoter assay (gray bars). (**C**) Cells were transfected with either scrambled or PKCδ siRNA and then incubated with 3 U/mL thrombin for 6 h. Protein levels of PKCδ, COX-2, and β-actin were determined via Western blot. (**D**) Cells were transfected with either scrambled or PKCδ siRNA and then incubated with 3 U/mL thrombin for the indicated time intervals. Phospho-PKCδ, total PKCδ, and β-actin levels were determined via Western blot. (**E**) Cells were pretreated with Rottlerin (1 µM) for 1 h and then incubated with 3 U/mL thrombin for 6 h. PGE_2_ generation was measured. (**F**) Cells were transfected with either scrambled or PKCδ siRNA and then incubated with 3 U/mL thrombin for 6 h. PGE_2_ generation was measured. Data are presented as mean ± SEM of three independent experiments (*n* = 3). # *p* < 0.05 indicates a significant difference between compared groups.

In a prior study, we established that thrombin-induced up-regulation of COX-2 expression and PGE_2_ release in rat vascular smooth muscle cells is mediated through the activation of PKCδ [39]. Building on our recent research [18], we delved deeper into the impact of PKCδ on 3 U/mL thrombin-induced COX-2 expression. It is noteworthy that 3 U/mL thrombin, generated during coagulation reactions, is associated with various inflammatory lung diseases [11,12]. To test this hypothesis, we employed Rottlerin, a selective PKCδ inhibitor. As depicted in Figure 1A, pretreatment with Rottlerin significantly attenuated thrombin-induced COX-2 expression in HTSMCs. Moreover, Rottlerin also inhibited thrombin-induced COX-2 mRNA levels and promoter activity in these cells, as shown in Figure 1B. To further validate the role of PKCδ, we utilized PKCδ siRNA to down-regulate PKCδ protein expression. Transfection with PKCδ siRNA markedly inhibited thrombin-enhanced COX-2 protein levels, as demonstrated in Figure 1C. Additionally, we assessed PKCδ phosphorylation to explore its involvement in thrombin-triggered up-regulation of COX-2 expression. Western blot analysis revealed time-dependent PKCδ phosphorylation following thrombin stimulation (Figure 1D). Furthermore, transfection with PKCδ siRNA effectively abolished PKCδ phosphorylation induced by thrombin in HTSMCs (Figure 1D). COX-2 is responsible for converting AA into bioactive lipids, including PGE_2_, which serves as a marker of COX-2 activity [2]. In this study, we demonstrated that thrombin-induced PGE_2_ secretion was reduced through pretreatment with Rottlerin or transfection with PKCδ siRNA, as shown in Figure 1E,F. These collective results in HTSMCs indicate that PKCδ activation plays a pivotal role in modulating the up-regulation of COX-2 and subsequent PGE_2_ release triggered by thrombin.

### 2.2. Thrombin Enhances COX-2 Expression via PKCδ-Dependent Pyk2 Activation

**Figure 2:**
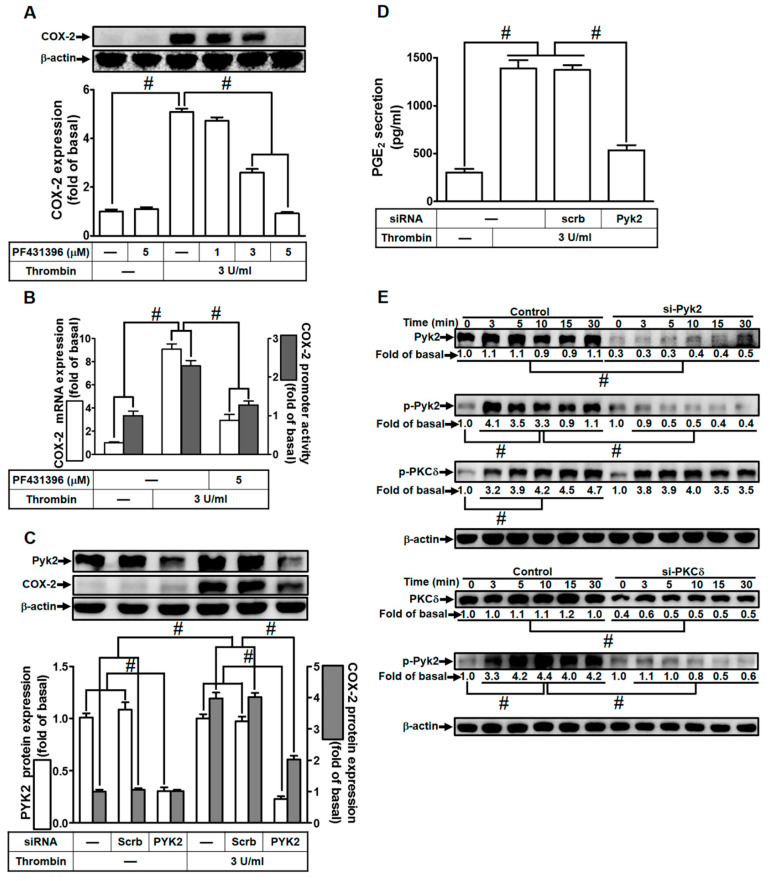
Thrombin induces COX-2 expression via Pyk2. (**A**) Cells were pretreated with PF431396 (1, 3, and 5 µM) for 1 h, followed by incubation with 3 U/mL thrombin for 6 h. The levels of COX-2 and β-actin proteins were assessed through Western blot analysis. (**B**) Cells were pretreated with PF431396 (5 µM) for 1 h and then exposed to 3 U/mL thrombin for 4 h. COX-2 mRNA levels were determined using real-time PCR (open bars), and COX-2 promoter activity was assessed through a promoter assay (gray bars). (**C**) Cells were transfected with either scrambled or Pyk2 siRNA and subsequently incubated with 3 U/mL thrombin for 6 h. Pyk2, COX-2, and β-actin protein levels were analyzed via Western blot. (**D**) Cells were transfected with either scrambled or Pyk2 siRNA and then treated with 3 U/mL thrombin for 6 h. PGE_2_ generation was quantified. (**E**) Cells were transfected with scrambled, Pyk2, or PKCδ siRNA, respectively, and then incubated with 3 U/mL thrombin for the specified time intervals. The levels of Pyk2, PKCδ, phospho-Pyk2, phospho-PKCδ, and β-actin were determined using Western blot analysis. The data represent the mean ± SEM of three independent experiments (*n* = 3). # *p* < 0.05 indicates a significant difference between the compared groups.

Wang and Reiser previously indicated that thrombin can induce Pyk2 phosphorylation via protease-activated receptor (PAR)-1 in rat brain astrocytes [24]. Additionally, in gonadotropin-releasing hormone-stimulated HEK293 cells, Pyk2 has been shown to be a downstream component of PKCδ activation [40]. Building upon these findings, we investigated the impact of Pyk2 on thrombin-induced COX-2 expression in HTSMCs. To test our hypothesis, we utilized PF431396, a selective Pyk2 inhibitor. As shown in Figure 2A, pretreatment with PF431396 significantly reduced thrombin-induced COX-2 expression in HTSMCs. Furthermore, PF431396 also inhibited thrombin-induced COX-2 mRNA levels and promoter activity, as depicted in Figure 2B. To confirm the role of Pyk2 in thrombin-induced COX-2 expression, we employed Pyk2 siRNA to down-regulate Pyk2 protein levels. Our results demonstrated that Pyk2 siRNA transfection effectively decreased thrombin-enhanced COX-2 protein expression (Figure 2C). Similarly, transfection with Pyk2 siRNA also led to a reduction in thrombin-induced PGE_2_ release (Figure 2D). Moving forward, we explored the relationship between Pyk2 and PKCδ phosphorylation in thrombin-enhanced COX-2 protein expression. We observed that thrombin induced Pyk2 phosphorylation in a time-dependent manner in HTSMCs, and this effect was attenuated by transfection with either Pyk2 or PKCδ siRNA (Figure 2E). Notably, Pyk2 siRNA transfection did not significantly impact PKCδ phosphorylation stimulated by thrombin (Figure 2E), suggesting that Pyk2 operates downstream of PKCδ signaling. In summary, our findings indicate that in HTSMCs, PKCδ-dependent activation of Pyk2 plays a crucial role in modulating the up-regulation of COX-2/PGE_2_ stimulated by thrombin.

### 2.3. Thrombin Up-Regulates COX-2 Expression through c-Src Activation

**Figure 3:**
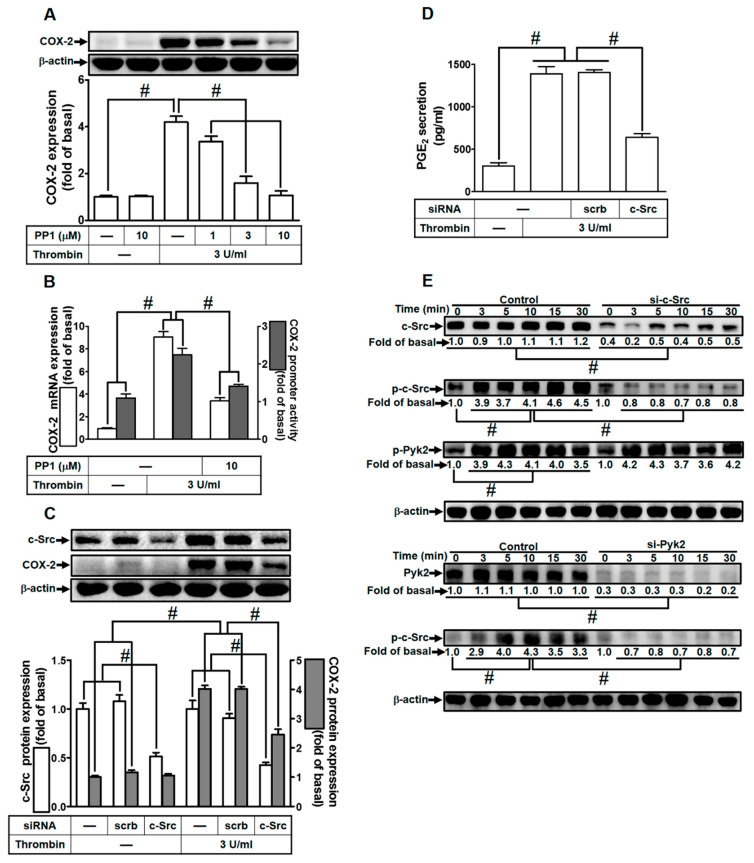
Thrombin induces COX-2 expression via c-Src. (**A**) Cells were pretreated with PP1 (1, 3, and 10 µM) for 1 h and then exposed to 3 U/mL thrombin for 6 h. COX-2 and β-actin protein levels were assessed through Western blot analysis. (**B**) Cells were pretreated with PP1 (10 µM) for 1 h, followed by incubation with 3 U/mL thrombin for 4 h. COX-2 mRNA levels were determined using real-time PCR (open bars), and COX-2 promoter activity was assessed through a promoter assay (gray bars). (**C**) Cells were transfected with either scrambled or c-Src siRNA and subsequently exposed to 3 U/mL thrombin for 6 h. Protein levels of c-Src, COX-2, and β-actin were analyzed via Western blot. (**D**) Cells were transfected with either scrambled or c-Src siRNA and then treated with 3 U/mL thrombin for 6 h. PGE_2_ generation was quantified. (**E**) Cells were transfected with scrambled, c-Src, or Pyk2 siRNA, respectively, and then exposed to 3 U/mL thrombin for the specified time intervals. Levels of c-Src, Pyk2, phospho-c-Src, phospho-Pyk2, and β-actin were determined using Western blot analysis. The data represent the mean ± SEM of three independent experiments (*n* = 3). # *p* < 0.05 indicates a significant difference between the compared groups.

Prior research has established that thrombin can trigger COX-2 expression and PGE_2_ release via c-Src activation in human neonatal ventricular cardiomyocytes [28]. Consequently, we delved into understanding the influence of c-Src on thrombin-induced responses in HTSMCs. HTSMCs were either pretreated with a c-Src inhibitor (PP1) or transfected with c-Src siRNA. As demonstrated in Figure 3A, pretreatment with PP1 significantly attenuated thrombin-induced COX-2 expression in HTSMCs. Additionally, PP1 hindered thrombin-induced COX-2 mRNA levels and promoter activity (Figure 3B). To validate c-Src’s role in thrombin-induced COX-2 expression, c-Src siRNA was employed to diminish c-Src protein levels. Figure 3C illustrates that c-Src siRNA transfection effectively reduced c-Src protein expression and markedly suppressed thrombin-enhanced COX-2 protein levels. Similarly, transfection with c-Src siRNA also diminished thrombin-induced PGE_2_ release (Figure 3D). We further investigated the connection between c-Src and Pyk2 phosphorylation in thrombin-mediated responses. Our results indicated that thrombin induced c-Src phosphorylation in a time-dependent manner, and this effect was mitigated through transfection with either c-Src or Pyk2 siRNA in HTSMCs (Figure 3E). However, c-Src siRNA transfection did not significantly impact Pyk2 phosphorylation stimulated by thrombin (Figure 3E), suggesting that c-Src operates downstream of Pyk2. In summary, these findings suggest that in HTSMCs, Pyk2-dependent c-Src activation plays a crucial role in the up-regulation of COX-2 and PGE_2_ induced by thrombin.

### 2.4. Thrombin Induces COX-2 Expression through EGFR Activation

**Figure 4:**
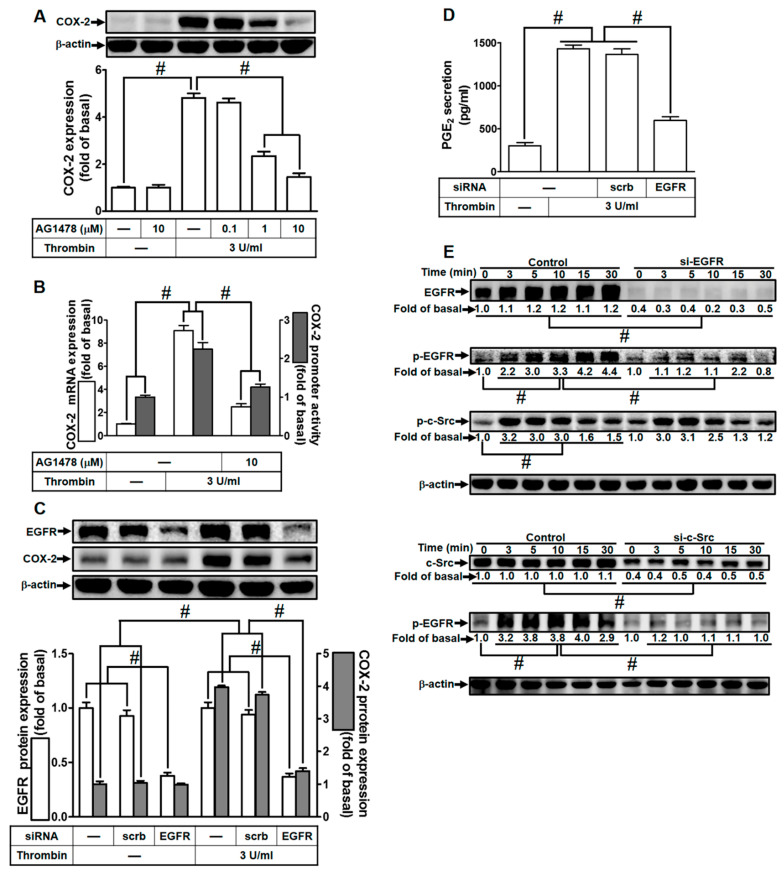
Thrombin induces COX-2 expression via EGFR. (**A**) Cells were pretreated with AG1478 (0.1, 1, and 10 µM) for 1 h and subsequently exposed to 3 U/mL thrombin for 6 h. COX-2 and β-actin protein levels were assessed using Western blot analysis. (**B**) Cells were pretreated with AG1478 (10 µM) for 1 h, followed by incubation with 3 U/mL thrombin for 4 h. COX-2 mRNA levels were determined using real-time PCR (open bars), and COX-2 promoter activity was assessed through a promoter assay (gray bars). (**C**) Cells were transfected with either scrambled or EGFR siRNA and then incubated with 3 U/mL thrombin for 6 h. Protein levels of EGFR, COX-2, and β-actin were analyzed via Western blot. (**D**) Cells were transfected with either scrambled or EGFR siRNA and then treated with 3 U/mL thrombin for 6 h. PGE_2_ generation was quantified. (**E**) Cells were transfected with scrambled, EGFR, or c-Src siRNA, respectively, and then exposed to 3 U/mL thrombin for the indicated time intervals. Levels of EGFR, c-Src, phospho-EGFR, phospho-c-Src, and β-actin were determined using Western blot analysis. The data represent the mean ± SEM of three independent experiments (*n* = 3). # *p* < 0.05 indicates a significant difference between the compared groups.

In our previous investigations, we established EGFR as a downstream component transactivated by c-Src, leading to the expression of inflammatory proteins across various cell types [23,28]. To delve deeper into the role of EGFR in thrombin-induced responses, we utilized a selective EGFR inhibitor (AG1478) or EGFR siRNA. As illustrated in Figure 4A, pretreatment with AG1478 significantly attenuated thrombin-induced COX-2 expression in HTSMCs. AG1478 also inhibited thrombin-induced COX-2 mRNA levels and promoter activity, as shown in Figure 4B. To further validate EGFR’s involvement, we employed EGFR siRNA to down-regulate EGFR expression. The down-regulation of EGFR expression through siRNA transfection impeded thrombin-induced COX-2 up-regulation, as demonstrated in Figure 4C. Consistently, transfection with EGFR siRNA also reduced thrombin-induced PGE_2_ release, as depicted in Figure 4D. Furthermore, we investigated the relationship between EGFR and c-Src phosphorylation in thrombin-induced responses. Thrombin time-dependently stimulated EGFR phosphorylation, which was diminished through transfection with either EGFR siRNA or c-Src siRNA (Figure 4E). Importantly, EGFR siRNA did not significantly affect thrombin-stimulated c-Src phosphorylation (Figure 4E), indicating that EGFR functions downstream of c-Src signaling. In summary, our findings highlight the crucial role of c-Src-dependent EGFR activation in modulating the up-regulation of COX-2 and PGE_2_ induced by thrombin in HTSMCs.

### 2.5. Thrombin Induces COX-2 Expression via PI3K/Akt

**Figure 5:**
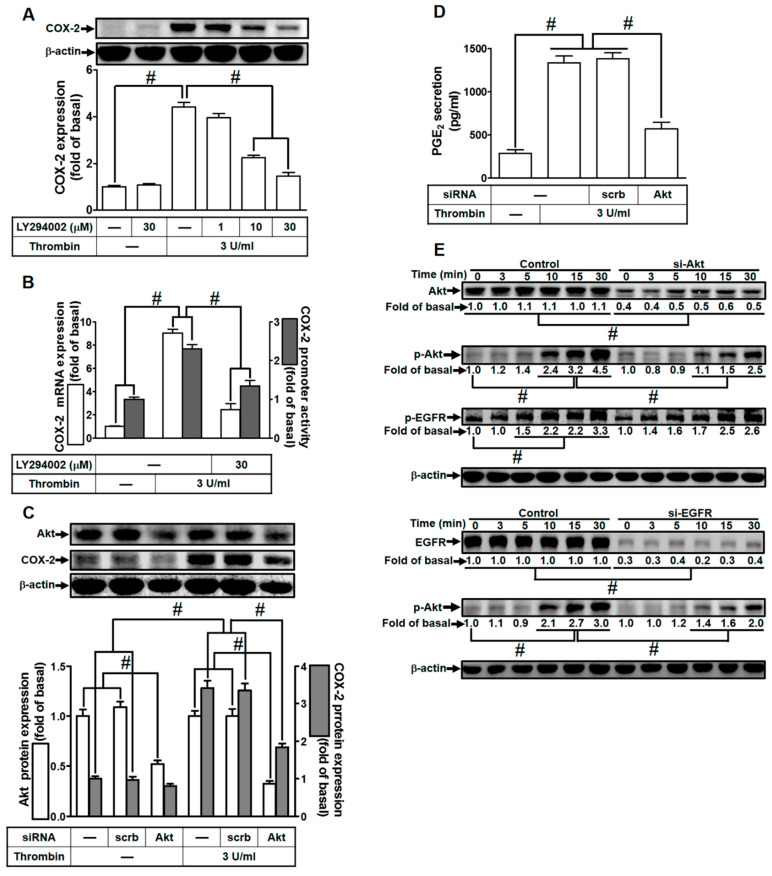
Thrombin induces COX-2 expression via PI3K/Akt. (**A**) Cells were pretreated with LY294002 (1, 10, and 30 µM) for 1 h, followed by incubation with 3 U/mL thrombin for 6 h. The levels of COX-2 and β-actin protein were assessed using Western blot analysis. (**B**) Cells were pretreated with LY294002 (30 µM) for 1 h and then exposed to 3 U/mL thrombin for 4 h. COX-2 mRNA levels were determined via real-time PCR (open bars), and the promoter activity of COX-2 was evaluated through promoter assay (gray bars). (**C**) Cells were transfected with either scrambled or Akt siRNA and then incubated with 3 U/mL thrombin for 6 h. The protein levels of Akt, COX-2, and β-actin were measured using Western blot analysis. (**D**) Cells were transfected with either scrambled or Akt siRNA and then incubated with 3 U/mL thrombin for 6 h. PGE_2_ generation was quantified. (**E**) Cells were transfected with scrambled, Akt, or EGFR siRNA, respectively, and then treated with 3 U/mL thrombin for the indicated time intervals. The levels of Akt, EGFR, phospho-Akt, phospho-EGFR, and β-actin were analyzed using Western blot. Data are presented as mean ± SEM of three independent experiments (*n* = 3). # *p* < 0.05 indicates a significant difference between the compared groups.

In our earlier research, we established the involvement of PI3K/Akt activation in the up-regulation of COX-2 expression and PGE_2_ release induced by thrombin in human neonatal ventricular cardiomyocytes [28]. Furthermore, EGFR has been identified as an upstream component of PI3K/Akt signaling. Thus, we delved into the role of PI3K/Akt in thrombin-mediated responses in HTSMCs. For this purpose, HTSMCs were either pretreated with LY294002, a selective PI3K inhibitor, or transfected with Akt siRNA. As depicted in Figure 5A, pretreatment with LY294002 resulted in a reduction in thrombin-induced COX-2 expression. Additionally, LY294002 inhibited thrombin-induced COX-2 mRNA levels and promoter activity (Figure 5B). To further elucidate the critical role of Akt in thrombin-induced COX-2 expression, we employed Akt siRNA to knock down Akt protein expression. This approach significantly inhibited thrombin-enhanced COX-2 protein levels (Figure 5C). Consistently, transfection with Akt siRNA also reduced thrombin-induced PGE_2_ release (Figure 5D). Furthermore, we explored the relationship between Akt and EGFR phosphorylation in thrombin-induced responses. Thrombin time-dependently stimulated Akt phosphorylation in HTSMCs, and this effect was attenuated through transfection with either Akt siRNA or EGFR siRNA (Figure 5E). However, transfection with Akt siRNA did not significantly affect EGFR phosphorylation stimulated by thrombin (Figure 5E), indicating that Akt functions downstream of EGFR signaling. In summary, these findings suggest that in HTSMCs, EGFR-dependent PI3K/Akt activation plays a pivotal role in modulating the up-regulation of COX-2 and PGE_2_ induced by thrombin.

### 2.6. Thrombin Induces COX-2 Expression via AP-1

**Figure 6:**
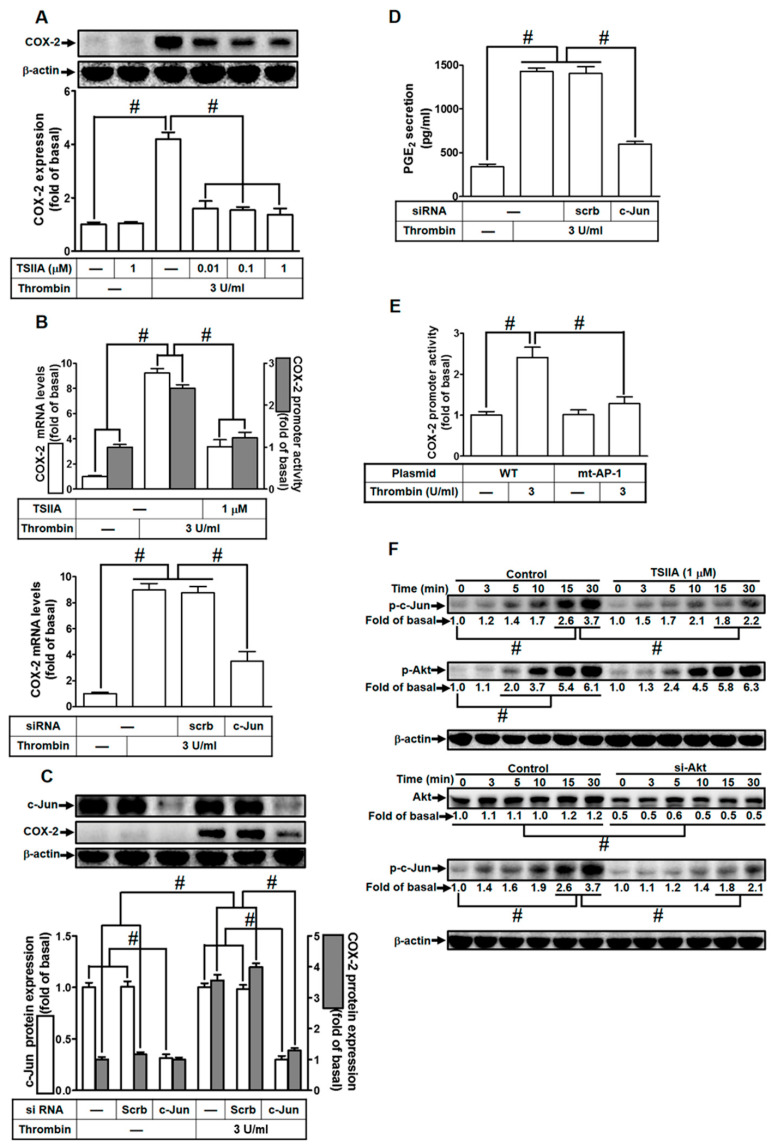
Thrombin induces COX-2 expression via AP-1. (**A**) Cells were preincubated with varying concentrations of Tanshinone IIA (0.01, 0.1, and 1 µM) for 1 h, followed by stimulation with 3 U/mL thrombin for 6 h. Western blot analysis was performed to assess the levels of COX-2 and β-actin proteins. (**B**) Cells were preincubated with Tanshinone IIA (1 µM) for 1 h and then stimulated with 3 U/mL thrombin for 4 h (upper panel). Real-time PCR was utilized to measure COX-2 mRNA levels (open bars), and promoter activity was evaluated through promoter assay (gray bars). Cells were transfected with either scrambled or c-Jun siRNA and subsequently treated with 3 U/mL thrombin for 4 h (bottom panel). COX-2 mRNA levels were determined via real-time PCR. (**C**) Cells were transfected with scrambled or c-Jun siRNA and then treated with 3 U/mL thrombin for 6 h. Western blot analysis was performed to assess the levels of c-Jun, COX-2, and β-actin proteins. (**D**) Cells were transfected with scrambled or c-Jun siRNA and then exposed to 3 U/mL thrombin for 6 h. PGE_2_ generation was quantified. (**E**) Cells were transfected with wild-type COX-2 promoter or mutated AP-1 binding site COX-2 promoter and then stimulated with 3 U/mL thrombin for 4 h. Promoter activity of COX-2 was determined using promoter assays. (**F**) Cells were either preincubated without or with Tanshinone IIA (1 µM) for 1 h, or transfected with scrambled or c-Jun siRNA, respectively. Subsequently, cells were stimulated with 3 U/mL thrombin for the indicated time intervals. Western blot analysis was performed to assess the protein levels of phospho-c-Jun, phospho-Akt, Akt, and β-actin. The data represent the mean ± SEM of three independent experiments (*n* = 3). # *p* < 0.05 indicates a significant difference between the compared groups.

In a study by Khanal et al., it was previously reported that genipin induces COX-2 expression through the activation of AP-1 and NF-κB in RAW 264.7 cells [29]. AP-1 activation is known to occur through various signaling pathways, including PKCδ [30], c-Src [23], and PI3K/Akt [23]. Therefore, we delved into the role of AP-1 in thrombin-induced COX-2 expression and sought to determine its involvement in this process. To investigate this hypothesis, HTSMCs were pretreated with Tanshinone IIA, a selective AP-1 inhibitor, or transfected with c-Jun siRNA (c-Jun being a subunit of AP-1). As shown in Figure 6A, pretreatment with Tanshinone IIA significantly reduced thrombin-induced COX-2 expression in HTSMCs. Additionally, Tanshinone IIA inhibited thrombin-induced COX-2 mRNA levels and promoter activity (Figure 6B). Furthermore, the role of c-Jun in thrombin-induced COX-2 expression was confirmed through transfecting cells with c-Jun siRNA, leading to a reduction in thrombin-induced COX-2 mRNA levels (Figure 6B). To establish the critical role of AP-1 in thrombin-induced COX-2 expression, c-Jun protein expression was knocked down using c-Jun siRNA. As depicted in Figure 6C, transfection with c-Jun siRNA significantly inhibited thrombin-enhanced COX-2 protein levels. Moreover, this treatment also reduced thrombin-induced PGE_2_ release (Figure 6D). The interaction between AP-1 and the COX-2 promoter was further examined through measuring thrombin-stimulated COX-2 promoter activity in HTSMCs transfected with a point-mutated AP-1 binding site on the COX-2 promoter. This experiment revealed that AP-1 binding to the COX-2 promoter was necessary for thrombin-induced COX-2 expression (Figure 6E). To understand how AP-1 phosphorylation regulates the up-regulation of COX-2 by thrombin, c-Jun phosphorylation was assessed via Western blot. Pretreatment with Tanshinone IIA inhibited time-dependent thrombin-induced AP-1 phosphorylation (Figure 6F). Furthermore, thrombin-stimulated AP-1 phosphorylation was reduced via pretreatment with Tanshinone IIA or transfection with Akt siRNA. Interestingly, Akt phosphorylation was not affected by Tanshinone IIA pretreatment, indicating that AP-1 operates downstream of Akt in thrombin-mediated responses (Figure 6F). In conclusion, these findings suggest that in HTSMCs, Akt-dependent AP-1 activation is involved in thrombin-induced COX-2 expression and is associated with PGE_2_ release.

### 2.7. Resveratrol Blocks Protein Kinase Activation to Attenuate Thrombin-Induced COX-2 Expression and PGE_2_ Secretion

**Figure 7:**
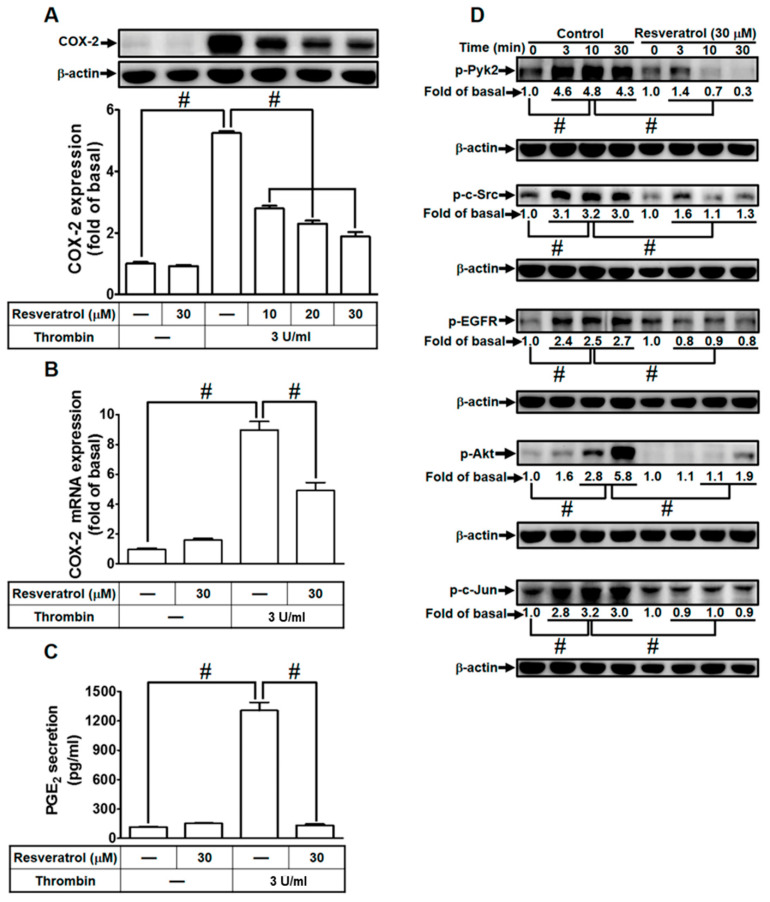
Resveratrol mitigates thrombin-induced COX-2 expression and PGE_2_ secretion through impeding protein kinase activation. (**A**) Cells were pretreated with resveratrol (10, 20, and 30 µM) for 1 h, followed by incubation with 3 U/mL thrombin for 6 h. Western blot analysis was performed to determine COX-2 and β-actin protein levels. (**B**) Pretreatment with resveratrol (30 µM) for 1 h, followed by incubation with 3 U/mL thrombin for 4 h, resulted in decreased COX-2 mRNA levels, as assessed via real-time PCR. (**C**) Cells, pretreated with resveratrol (30 µM) for 1 h, were incubated with 3 U/ml thrombin for 6 h. PGE_2_ levels in the media were measured using ELISA. (**D**) Pretreatment with 30 µM resveratrol for 1 h, followed by incubation with 3 U/mL thrombin for various time intervals, showed reduced levels of phospho-Pyk2, phospho-c-Src, phospho-EGFR, phospho-Akt, and phospho-c-Jun, as determined via Western blot with β-actin serving as an internal control. The data represent the mean ± SEM of three independent experiments (*n* = 3). # *p* < 0.05, indicating a significant difference between the compared groups.

In our previous research, we established that resveratrol effectively dampens *Staphylococcus aureus* (*S. aureus*)-induced inflammatory signaling through diminishing the activity of key elements such as c-Src, PDGFR, JNK1/2, p38 MAPK, and AP-1 in human pulmonary alveolar epithelial cells [34]. Additionally, our investigations revealed that resveratrol mitigates COX-2 expression through inhibiting the phosphorylation and acetylation of p65, c-Jun, and Fos in human rheumatoid arthritis synovial fibroblasts [35]. In light of these findings, we delved into exploring whether resveratrol could regulate protein kinase activities to curb thrombin-induced inflammatory responses in HTSMCs. Initially, we observed that pre-treatment with resveratrol dose-dependently suppressed thrombin-induced COX-2 protein levels and mRNA expression in HTSMCs (Figure 7A,B). Moreover, resveratrol also curtailed thrombin-induced PGE_2_ release (Figure 7C). We further delved into understanding whether the anti-inflammatory effects of resveratrol on thrombin-mediated responses were attributed to the inhibition of specific protein kinases. Our results demonstrated that pretreatment with resveratrol mitigated thrombin-stimulated phosphorylation of Pyk2, c-Src, EGFR, Akt, and c-Jun, while leaving PKCδ unaffected, in HTSMCs (Figure 7D). These findings indicate that in HTSMCs, resveratrol modulates thrombin-induced COX-2 expression and PGE_2_ release, at least in part, through restraining the activation of Pyk2, c-Src, EGFR, Akt, and c-Jun.

## 3. Discussion

**Figure 8:**
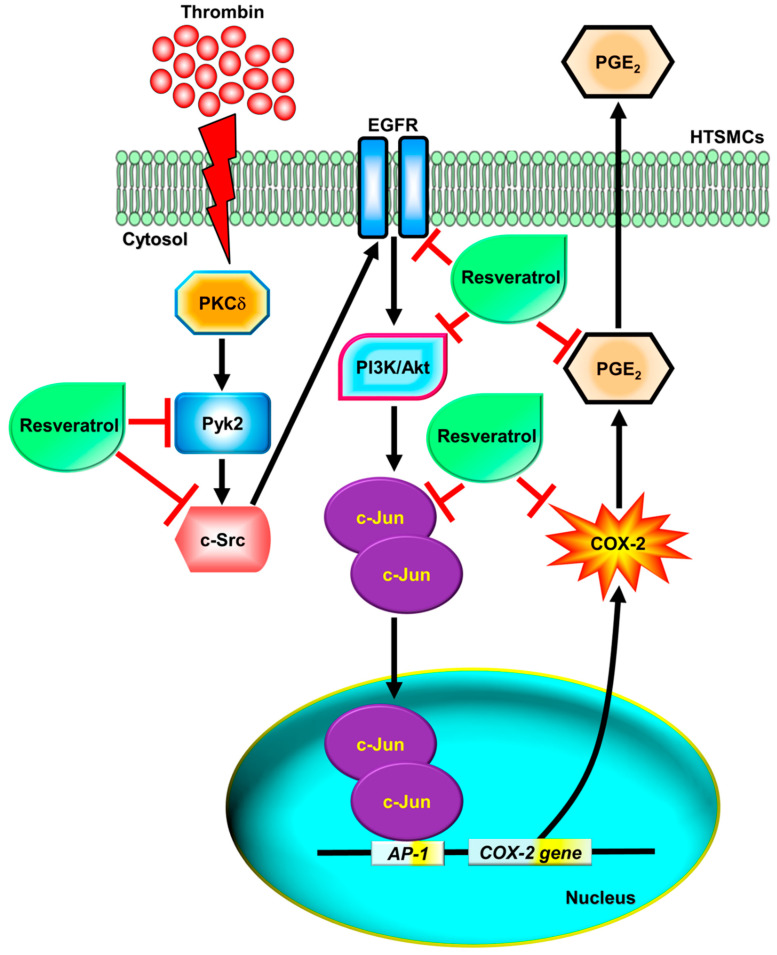
Schematic diagram illustrating the proposed signaling pathways involved in thrombin-induced COX-2 expression and PGE_2_ secretion, which are inhibited by resveratrol in HTSMCs. Thrombin induces COX-2 expression and PGE_2_ generation through a PKCδ/Pyk2/c-Src/EGFR/PI3K/Akt-dependent pathway, leading to AP-1 activation in HTSMCs. Resveratrol inhibits thrombin-induced COX-2 expression and PGE_2_ generation through blocking the activation of Pyk2, c-Src, EGFR, PI3K/Akt, and c-Jun.

COX-2 plays a crucial role in generating proinflammatory eicosanoids and is a key mediator in airway inflammatory responses [5]. Numerous studies indicate that the COX-2/PGE_2_ signaling pathway is highly involved in lung inflammatory disorders, while also contributing to normal airway physiology, such as airway adaptation during physical exercise [4,6,9]. Additionally, PGE_2_ not only promotes proinflammatory effects but also exhibits anti-inflammatory activity through the EP4 receptor in the lungs [8]. The exact mechanisms through which thrombin induces COX-2/PGE_2_ expression and how this process is counteracted by resveratrol remain incompletely understood in HTSMCs. In Figure 8, our research confirmed that thrombin-induced COX-2/PGE_2_ expression occurs through a complex signaling pathway involving PKCδ/Pyk2/c-Src/EGFR/PI3K/Akt/AP-1. Silencing genes via siRNA targeting PKCδ, Pyk2, c-Src, EGFR, Akt, or c-Jun, as well as pretreatment with inhibitors specific to these proteins, effectively halted thrombin-induced COX-2 expression and PGE_2_ synthesis. Furthermore, resveratrol inhibited the phosphorylation of Pyk2, c-Src, EGFR, Akt, and c-Jun, thus suppressing thrombin-induced COX-2 expression and PGE_2_ release in HTSMCs.

Thrombin, a key procoagulant serine protease released in response to tissue injury, is not only critical for maintaining hemostasis but also exerts a multifaceted impact on cellular responses. Its influence spans a wide array of biological processes, including inflammation and immune responses, making it a pivotal player in the intricate web of physiological regulation [10]. In the context of respiratory health, previous studies have unveiled thrombin’s significant involvement in inflammation. Specifically, thrombin has been linked to the recruitment of neutrophils and the escalation of chemokine levels in the airways, as evidenced in studies conducted on mice treated with thrombin [41]. This proinflammatory effect of thrombin is mediated through the activation of protease-activated receptors (PARs), a family of G-protein-coupled receptors (GPCRs) [42]. Through PARs, thrombin not only amplifies the release of crucial proinflammatory cytokines like interleukin (IL)-6, IL-8, C-C motif chemokine ligand 2 (CCL2), IL-1β, and tumor necrosis factor (TNF)-α but also enhances the production of the prostanoid PGE_2_ within the respiratory system [43]. Intriguingly, recent research has elucidated the intricate molecular mechanisms underlying thrombin-induced inflammation. Specifically, our studies have delineated that thrombin promotes COX-2 expression and PGE_2_ synthesis through the activation of PAR1/Gq or Gi/o-dependent cascades [18]. These findings not only emphasize the pivotal role of thrombin in airway inflammatory diseases but also pinpoint the PAR1 receptor as a potential therapeutic target for advanced interventions in airway inflammation. Through understanding the precise mechanisms through which thrombin orchestrates these inflammatory responses, novel avenues for therapeutic exploration emerge, holding the promise of more targeted and effective interventions in the realm of respiratory health.

PKCs, a subset of AGC kinases alongside cyclic AMP-regulated kinases and cyclic GMP-regulated kinases, are pivotal regulators of inflammatory responses, gene expression, and cell proliferation. These kinases are categorized into three groups based on their structural requirements for additional cofactors and calcium [44]. Within this family, PKC isoforms have been closely linked to the regulation of airway smooth muscle growth and contractility, highlighting their significance in respiratory physiology [45]. Particularly, PKCδ, a specific isoform of PKCs, has been implicated in diverse cellular functions, including the control of differentiation, growth, and migration [46]. Its role in lung infections has been illuminated, and it has been proposed as a marker of lung inflammation [47]. In our previous research, we established that thrombin-induced COX-2 expression and PGE_2_ release in rat vascular smooth muscle cells are mediated through the activation of PKCδ [39]. These findings align seamlessly with our current observations, underscoring the pivotal role of PKCδ in thrombin-induced COX-2 expression and PGE_2_ secretion. To further validate this hypothesis, we conducted experiments involving the pretreatment of cells with Rottlerin, a specific PKCδ inhibitor, and the transfection of cells with PKCδ siRNA. Both interventions significantly attenuated the responses induced by thrombin in HTSMCs, providing robust evidence for the crucial involvement of PKCδ in these cellular processes.

The intricate interplay of molecular signaling pathways involving focal adhesion tyrosine kinases FAK and Pyk2, as well as the protein tyrosine kinase c-Src, serves as a fundamental regulatory mechanism governing various cellular processes. FAK and Pyk2, pivotal mediators strategically positioned within cells, orchestrate complex signaling cascades essential for cell migration, proliferation, and survival [24]. Pyk2, activated by diverse GPCR ligands and inflammatory cytokines, triggers c-Src kinase activity, forming a critical nexus in cellular responses [22]. In our comprehensive studies with HTSMCs, we meticulously delineated how thrombin, a multifaceted procoagulant serine protease, stimulates COX-2 expression and PGE_2_ release through Pyk2 activation. In the intricate landscape of cellular responses, exposure of acinar cells to PMA revealed heightened phosphorylation of PKCδ and Pyk2, underscoring their dynamic involvement in signaling events [25]. Intriguingly, inhibiting PKCδ did not merely dampen thrombin-induced Pyk2 phosphorylation. This finding underscores a nuanced mechanism wherein PKCδ orchestrates the activation of Pyk2, establishing a pivotal link between these kinases in mediating thrombin-induced COX-2 expression and subsequent PGE_2_ synthesis. This intricate interplay between PKCδ and Pyk2 highlights their cooperative role in airway inflammatory diseases, shedding light on potential therapeutic targets for intervention. Furthermore, c-Src, a member of the highly conserved Src family of protein tyrosine kinases, occupies a central position in cellular responses, participating in diverse processes such as innate immune responses and signaling cascades induced by cytokines, antigens, and growth factors [2]. The thrombin-induced activation of c-Src has been a subject of scientific exploration, and our investigations reaffirm this phenomenon [23]. Through employing specific siRNA to knock down c-Src, we deciphered the essential role played by this kinase. Its down-regulation significantly attenuated PGE_2_ release induced by thrombin, thereby inhibiting COX-2 expression. This observation underscores the pivotal role of thrombin-induced c-Src phosphorylation in amplifying inflammation in response to this protease. In essence, our findings unravel a complex network of interactions involving PKCδ, Pyk2, and c-Src, shedding light on the intricate mechanisms underlying thrombin-induced COX-2 expression and PGE_2_ synthesis. These insights not only deepen our understanding of airway inflammatory responses but also pave the way for innovative therapeutic strategies targeting these specific pathways, holding promise for mitigating inflammatory disorders.

Numerous studies have elucidated how various GPCR ligands activate the Akt pathway through transactivating EGFR or PDGFR in different cell types [2]. However, the mechanisms through which thrombin initiates COX-2 expression, particularly through the c-Src-dependent transactivation of EGFR in HTSMCs, remain poorly understood. In our comprehensive investigation, we uncovered that thrombin robustly induces COX-2 expression via an EGFR-dependent pathway. This assertion was strongly supported by our experimental data, where the use of an EGFR inhibitor or EGFR-specific siRNA clearly suppressed this effect. Additionally, our research in these cells highlighted the pivotal role of c-Src in mediating thrombin-induced EGFR transactivation. Thus, thrombin effectively triggers COX-2 expression and subsequent PGE_2_ release through a precisely orchestrated signaling cascade involving PKCδ/Pyk2/c-Src, culminating in EGFR activation. Recent studies have emphasized the crucial involvement of various components of the PI3K pathway in the expression and activation of inflammatory mediators, inflammatory cell recruitment, and immune cell function. Growth factors like EGF and PDGF are well-established activators of Akt, a process that can be effectively blocked using agents such as Wortmannin, LY294002, or through the overexpression of dominant-negative mutants of PI3K [48]. Akt, a central player in the pathogenesis of inflammatory responses, emerges as a key node in this intricate network [2]. Our experimental findings demonstrated that inhibiting the PI3K/Akt pathway, achieved through targeted down-regulation of Akt using specific siRNA, significantly mitigated the thrombin-induced release of PGE_2_. This effect was attributed to the simultaneous down-regulation of COX-2 expression. Furthermore, our study firmly established that thrombin stimulates Akt activation via a precisely coordinated PKCδ/Pyk2/c-Src/EGFR pathway, ultimately culminating in the induction of COX-2 expression in HTSMCs. These insights not only deepen our comprehension of the molecular intricacies governing airway inflammatory responses but also provide promising avenues for therapeutic interventions targeting these specific signaling pathways. Such interventions hold potential in the management of inflammatory disorders. Inflammatory responses triggered by extracellular stimuli heavily rely on the activation of AP-1, a transcription factor that crucially regulates the expression of various target genes. The COX-2 promoter contains diverse binding sequences for different transcription factors, including AP-1, emphasizing its pivotal role in regulating COX-2-dependent inflammatory responses [29,49]. Our research has unequivocally demonstrated that inhibiting AP-1 effectively reduces thrombin-induced COX-2 expression. Moreover, our studies revealed the thrombin-induced phosphorylation of c-Jun in these cells. AP-1 activation has been documented through multiple signaling pathways, including PKCδ [30], Pyk2 [23], c-Src [23], or PI3K/Akt [23]. Our current findings align with these pathways, indicating that in HTSMCs, thrombin stimulates c-Jun phosphorylation through a precisely orchestrated PKCδ/Pyk2/EGFR/PI3K/Akt pathway. These insights deepen our understanding of the intricate mechanisms underlying inflammatory responses and offer potential therapeutic targets for inflammatory disorders.

Resveratrol, a natural polyphenol and phytoalexin, has garnered significant interest over the last decade because of its diverse therapeutic properties, including its potential as an anticancer, anti-inflammatory, and anti-oxidant agent [50,51]. For instance, Donnelly et al. demonstrated that resveratrol inhibits cytokine-induced expression of inducible nitric oxide synthase, nitrite production, COX-2 expression, and the release of granulocyte-macrophage colony-stimulating factor and IL-8 in human primary airway epithelial cells [38]. Culpitt et al. reported its ability to suppress the release of inflammatory cytokines from alveolar macrophages isolated from COPD patients [37]. Moreover, our recent study indicated that resveratrol potentially dampens the inflammatory response induced by *S. aureus* through modulating signaling pathways, including c-Src, PDGFR, JNK1/2, p38 MAPK, and AP-1 activation. This regulation leads to reduced expression of vascular cell adhesion molecule-1 (VCAM-1) and decreased monocyte adhesion [34]. Additionally, in human rheumatoid arthritis synovial fibroblasts, resveratrol has been shown to attenuate COX-2 expression induced by bradykinin through the regulation of AP-1 and NF-κB activation [35]. These findings highlight resveratrol’s potential as a therapeutic agent in mitigating inflammatory responses. Furthermore, the complexities of cellular responses and the diverse pathways involved in different cell types and disease contexts contribute to the variability observed in the effects of resveratrol. While resveratrol has shown consistent anti-inflammatory properties in various studies [50,51], the specific mechanisms can vary due to the intricate interplay of signaling pathways, cellular environments, and the nature of the inflammatory stimulus. Moreover, resveratrol’s multifaceted actions highlight its potential as a therapeutic agent. Its ability to interfere with specific pathways, such as AP-1 activation, suggests a targeted approach to mitigate inflammation. Additionally, its impact on apoptosis and cancer cells underscores its potential in cancer therapy. The nuanced effects of resveratrol underscore the importance of conducting comprehensive studies to elucidate its precise mechanisms of action in different cellular contexts. In summary, resveratrol’s modulation of inflammatory responses and its potential in cancer therapy are promising areas of research. Continued investigations are essential to unravel the intricate details of resveratrol’s actions, paving the way for the development of targeted therapies and enhancing our understanding of its therapeutic potential in diverse disease conditions.

## 4. Materials and Methods

### 4.1. Materials

Dulbecco’s modified Eagle’s medium (DMEM)/F-12 medium, fetal bovine serum (FBS), and TRIzol reagent were procured from Invitrogen (Carlsbad, CA, USA). Hybond C membrane and the enhanced chemiluminescence (ECL) Western blot detection system were obtained from GE Healthcare Biosciences (Buckinghamshire, England, UK). Antibodies against phosphorylated proteins, including anti-phospho-PKCδ (#9374, Thr505), anti-phospho-Pyk2 (#3291, Tyr402), anti-phospho-c-Src (#2101, Tyr416), anti-phospho-EGFR (#4407, Tyr1173), anti-phospho-Akt (#9271, Ser473), anti-phospho-c-Jun (#2361, Ser63), and anti-COX-2 (#12282), were sourced from Cell Signaling (Danvers, MA, USA). Antibodies against non-phosphorylated proteins, such as anti-PKCδ (sc-213), anti-c-Src (sc-18), anti-EGFR (sc-03-G), anti-Akt (sc-8312), and anti-c-Jun (sc-44), were obtained from Santa Cruz (Santa Cruz, CA, USA). The antibody against Pyk2 (ab55358) was procured from Abcam (Cambridge, MA, USA). The anti-glyceraldehyde-3-phosphate dehydrogenase (GAPDH) (mouse monoclonal antibody, Cat# MCA-1D4) was sourced from EnCor Biotechnology (Gainesville, FL, USA). Chemical compounds, including Rottlerin, PF431396, PP1, AG1478, LY294002, and Tanshinone IIA, were supplied by Biomol (Plymouth Meeting, PA, USA). Supplies for sodium dodecyl sulfate-polyacrylamide gel electrophoresis (SDS-PAGE) were obtained from MDBio Inc. (Taipei, Taiwan). Thrombin (BRENDA: EC3.4.21.5, from bovine plasma, Cat# T4648), enzymes, and other chemicals were purchased from Sigma (St. Louis, MO, USA).

### 4.2. Cell Culture

HTSMCs were obtained from ScienCell Research Lab (San Diego, CA, USA) and cultured following the protocol described by Lee et al. [5]. All experiments were performed using HTSMCs from passages 4 to 8. Importantly, it is worth noting that the concentrations of the inhibitors used and the presence of DMSO did not have any cytotoxic effects on these cells. This was confirmed through the use of an XTT assay kit, although the specific data are not shown here.

### 4.3. Western Blot 

Cells in a state of growth arrest were exposed to thrombin at 37 °C for specified time intervals. Afterward, the cells were washed, scraped, collected, and centrifuged at 45,000× *g* at 4 °C for 1 h to obtain whole-cell extracts, following the methodology outlined by Lee et al. [5]. Subsequently, the samples were denatured, subjected to SDS-PAGE using a 10% running gel, and transferred onto a nitrocellulose membrane. These membranes were then incubated with antibodies, which were diluted in Tween–Tris-buffered saline (TTBS) to a range of 1:500–1000 for 24 h. This was followed by an additional 1 h of incubation with an anti-mouse horseradish peroxidase antibody. Immunoreactive bands were visualized using an ECL reagent, and the immunoblots were captured using a UVP BioSpectrum 500 imaging system (Upland, CA, USA). Densitometry analysis was performed using UN-SCAN-IT gel Version 7.1 software (Orem, UT, USA).

### 4.4. Real-Time PCR

Total RNA was extracted using TRIzol reagent, followed by reverse transcription into cDNA. Real-time PCR analysis was performed utilizing SYBR Green PCR reagents from Applied Biosystems (Branchburg, NJ, USA) with primers specific for COX-2 and GAPDH, following the protocol outlined by Yang et al. [18]. COX-2 expression levels were quantified through normalizing them to the expression levels of GAPDH. GAPDH is a commonly used housekeeping gene, allowing for standardization across different research studies. Normalization to GAPDH enhances the accuracy of gene expression quantification.

### 4.5. Measurement of PGE_2_ Generation

HTSMCs were grown in 6-well culture plates. When the cells reached confluence, they were exposed to thrombin for specific time periods at 37 °C. Afterward, the culture media were collected and stored at −80 °C until the assay was conducted. The levels of PGE_2_ were determined using a PGE_2_ enzyme-linked immunosorbent assay (ELISA) kit from Enzo Life Sciences (Lausen, Switzerland), following the manufacturer’s instructions.

### 4.6. Measurement of COX-2 Luciferase Promoter Activity

To create the COX-2-luc plasmid, we inserted the human COX-2 promoter region, which spans from −459 to +9 bp, into the pGL3-basic vector (Promega, Madison, WI, USA). The activity of COX-2-luc was assessed using a luciferase assay system (Promega, Madison, WI, USA), following the method outlined by Lee et al. [5]. Firefly luciferase activities were normalized against β-galactosidase (β-gal) activity.

### 4.7. Transient Transfection with Small Interfering RNAs (siRNAs)

Human siRNAs, specifically PKCδ (SASI_Hs01_00061170; NM_001354680.2), Pyk2 (SASI_Hs01_00032249; U33284.1), EGFR (SASI_Hs01_00215449; NM_001346941.2), c-Src (SASI_Hs01_00112905; NM_198291.3), Akt (SASI_Hs01_00105954; NM_001382431.1), and a scrambled control (negative control type 1) siRNA, were obtained from Sigma (St. Louis, MO, USA). Additionally, c-Jun siRNA (HSS105641, HSS105642, HSS180003; NM_002228.4) was sourced from Invitrogen Life Technologies (Carlsbad, CA, USA). These siRNAs were transiently transfected at a concentration of 100 nM using Lipofectamine™ RNAiMAX reagent, following the manufacturer’s instructions and the protocol detailed by Yang et al. [18].

### 4.8. Statistical Analysis

Statistical analysis was conducted using GraphPad Prism Program 6.0 software (GraphPad, San Diego, CA, USA). One-way ANOVA followed by Dunnett’s post hoc test was used for comparisons involving more than two data groups. In cases where multiple independent groups were compared, and normality assumptions for ANOVA were not met, the nonparametric Kruskal–Wallis test was employed, followed by Dunn’s post hoc test. Statistical significance was set at a threshold of *p* < 0.05. Post hoc tests were performed only when the F statistic yielded a *p* value less than 0.05, and homogeneity of variance was observed. All data were presented as the mean ± SEM, based on at least three separate experiments (*n* = number of independent cell culture preparations). Error bars were omitted if they fell within the symbol dimensions.

## 5. Conclusions

In summary, our findings elucidate the intricate signaling pathways in HTSMCs, where thrombin induces COX-2 expression and PGE_2_ release through a PKCδ/Pyk2/c-Src/EGFR/PI3K/Akt-dependent cascade, ultimately leading to AP-1 activation. Additionally, resveratrol mitigates the activation of key protein kinases, including Pyk2, c-Src, EGFR, Akt, and c-Jun, all of which are involved in thrombin-induced COX-2 expression and PGE_2_ release in HTSMCs. These results provide novel insights into the mechanisms underlying thrombin-induced COX-2 expression and PGE_2_ release, contributing to the escalation of inflammatory responses. A comprehensive understanding of the signaling mechanisms governing COX-2 gene regulation holds promise for the development of therapeutic strategies for anti-inflammatory interventions.

## Data Availability

The data presented in this study are available in present article.
